# Long-term Clinical Sequelae of Sexually Transmitted Infections in Women^1^

**DOI:** 10.3201/eid1011.040622_02

**Published:** 2004-11

**Authors:** Carolyn Deal, Ward Cates, Rosanna Peeling, Anna Wald

**Affiliations:** * National Institutes of Health, Bethesda, Maryland, USA;; † Family Health International, Durham, North Carolina, USA;; ‡ World Health Organization, Geneva, Switzerland;; § University of Washington, Seattle, Washington, USA

**Keywords:** sexually transmitted infections, STIs, women, conference summary

Sexually transmitted infections (STIs) are global and national health priorities because of theireffect on women and infants and the interrelationships with HIV and AIDS. STIs can cause a variety of long-term health problems. The sequelae of STIs include pelvic inflammatory disease (PID), infertility, tubal or ectopic pregnancy, cervical cancer, and perinatal or congenital infections in infants born to infected mothers.

## Gonorrhea and Chlamydia Control and Infertility Prevention

Infertility is one component in a spectrum of poor reproductive tract outcomes, such as ectopic pregnancy and chronic pelvic pain. Upper genital tract infections caused by STIs or poor gynecologic and obstetric care likely contribute to infertility. The role of PID in infertility and the "PID Iceberg" in the design of interventional strategies are presented. At the top of the iceberg are conditions that are visible, including symptomatic PID; at the base is asymptomatic PID, where the focus should be in trying to prevent ascending infections. A relationship exists between STIs and PID, as well as between PID and infertility; the etiology, however, is complex and involves interaction of the microbial, behavioral, and sociogeographic environments. A World Health Organization (WHO) study that involved 33 medical centers in 25 countries found evidence of high levels of STIs and tubal infertility in the same countries. A study in Sweden further contributed to understanding causality.

STI prevention is important in preventing infertility. Primary approaches can be built on safer behavior messages (similar to HIV messages), while secondary approaches can be broadened to have a wider application. Second, atypical or asymptomatic PID has a dominant influence. Early treatment of symptomatic PID can lower the risk of infertility.

## Syphilis

Disease and death associated with congenital syphilis are preventable. Twelve million new cases of syphilis are diagnosed each year. In Africa, 38% of women attending prenatal care receive syphilis screening. The disease accounts for 29% of perinatal deaths, 11% of neonatal deaths, and 26% of stillbirths.

A major barrier to prevention efforts is the lack of diagnostic tools that are effective in areas without electricity or running water. In 1996, with a grant from USAID and the Bill and Melinda Gates Foundation, rapid plasma reagin testing was decentralized in rural Haiti. Communities were provided with solar-powered batteries to run the centrifuge and rotator for the test. Training was provided to the workers, and quality control for same-day testing and treatment was implemented. The treatment rate improved from 41% to 100%, and the rate of congenital syphilis decreased by 75% during 2 years. The Sexually Transmitted Diseases Diagnostics Initiative, housed in the WHO, is promoting and facilitating the development, evaluation and application of tests that are affordable, sensitive, specific, user-friendly, rapid and robust, equipment-free, and deliverable to end users (ASSURED) and which would be appropriate for use in primary healthcare settings in developing countries.

## Herpes Simplex Virus (HSV) and HIV Acquisition

HSV-2 infection increases the risk of HIV acquisition. A meta-analysis of existing studies indicates that in an HSV-2–infected population, up to 50% of HIV infection is attributed to those with HSV-2. In the general population, with a 22% HSV-2 infection rate, 19% of HIV infection can be attributed to those with HSV-2 infections. A study in four urban areas of Africa found that higher HSV-2 rates were associated with higher rates of HIV. These epidemiologic data are strengthened by a biologic plausibility that HSV-2 affects HIV acquisition. HSV-2, as the most common cause of genital ulcers worldwide, possibly increases the risk of HIV transmission as well.

